# Crop Production under Drought and Heat Stress: Plant Responses and Management Options

**DOI:** 10.3389/fpls.2017.01147

**Published:** 2017-06-29

**Authors:** Shah Fahad, Ali A. Bajwa, Usman Nazir, Shakeel A. Anjum, Ayesha Farooq, Ali Zohaib, Sehrish Sadia, Wajid Nasim, Steve Adkins, Shah Saud, Muhammad Z. Ihsan, Hesham Alharby, Chao Wu, Depeng Wang, Jianliang Huang

**Affiliations:** ^1^National Key Laboratory of Crop Genetic Improvement, MOA Key Laboratory of Crop Ecophysiology and Farming System, College of Plant Science and Technology, Huazhong Agricultural UniversityWuhan, China; ^2^School of Agriculture and Food Sciences, The University of Queensland, GattonQLD, Australia; ^3^Department of Agronomy, University of AgricultureFaisalabad, Pakistan; ^4^College of Life Sciences, Beijing Normal UniversityBeijing, China; ^5^Department of Environmental Sciences, COMSATS Institute of Information TechnologyVehari, Pakistan; ^6^College of Horticulture, Northeast Agricultural University HarbinHarbin, China; ^7^Royal Wellington Golf ClubUpper Hutt, New Zealand; ^8^Cholistan Institute of Desert Studied, The Islamia University of BahawalpurBahawalpur, Pakistan; ^9^Department of Agronomy, The Islamia University of BahawalpurBahawalpur, Pakistan; ^10^Department of Biological Sciences, Faculty of Science, King Abdulaziz UniversityJeddah, Saudi Arabia; ^11^College of Life Science, Linyi UniversityLinyi, China; ^12^Hubei Collaborative Innovation Center for Grain Industry, Yangtze UniversityWuhan, China

**Keywords:** climate change, crop production, drought, heat stress, plant responses, stress management

## Abstract

Abiotic stresses are one of the major constraints to crop production and food security worldwide. The situation has aggravated due to the drastic and rapid changes in global climate. Heat and drought are undoubtedly the two most important stresses having huge impact on growth and productivity of the crops. It is very important to understand the physiological, biochemical, and ecological interventions related to these stresses for better management. A wide range of plant responses to these stresses could be generalized into morphological, physiological, and biochemical responses. Interestingly, this review provides a detailed account of plant responses to heat and drought stresses with special focus on highlighting the commonalities and differences. Crop growth and yields are negatively affected by sub-optimal water supply and abnormal temperatures due to physical damages, physiological disruptions, and biochemical changes. Both these stresses have multi-lateral impacts and therefore, complex in mechanistic action. A better understanding of plant responses to these stresses has pragmatic implication for remedies and management. A comprehensive account of conventional as well as modern approaches to deal with heat and drought stresses have also been presented here. A side-by-side critical discussion on salient responses and management strategies for these two important abiotic stresses provides a unique insight into the phenomena. A holistic approach taking into account the different management options to deal with heat and drought stress simultaneously could be a win-win approach in future.

## Introduction

Global food security is being haunted by the rapid increase in population and drastic changes in the climate (Lesk et al., 2016). In the wake of changing climate, drought, and heat stress have become the most important limiting factors to crop productivity and ultimately the food security. The reduced precipitation and changed rainfall patterns are causing the frequent onset of droughts around the world (Lobell et al., 2011). Severe droughts cause substantial decline in crop yields through negative impacts on plant growth, physiology, and reproduction (Yordanov et al., 2000; Barnabas et al., 2008). A recent study analyzed the data of studies published from 1980 to 2015 to report up to 21 and 40% yield reductions in wheat (*Triticum aestivum* L.) and maize (*Zea mays* L.), respectively due to drought on a global scale (Daryanto et al., 2016). Average global combined temperature of land and ocean surface has increased by 0.85°C between 1880 and 2012 (IPCC, 2014). An average increase of at least 0.2°C per decade is projected from now onward. The rising concentration of the greenhouse gasses is becoming a major cause of the global warming. Over the past 250 years a 30 and 150% rise in the concentration of the CO_2_ and methane has been observed (Lal, 2004; Friedlingstein et al., 2010). These stresses limit plant growth and productivity more than any other environmental factor. For instance, global wheat production was simulated to decline by 6% for each degree Celsius rise in temperature (Asseng et al., 2015). Although increasing temperatures are also beneficial for crop production in some cooler regions of the world, overall impact on global food security is still negative (Challinor et al., 2014).

Plants are subjected to the drought conditions when either the water supply to the roots is limited or the loss of water through transpiration is very high (Anjum et al., 2011). The severity of the damage caused by the drought is generally unpredictable as it is driven by various factors including, the rainfall patterns, moisture holding capacity of the soil, and water losses through evapotranspiration. Drought interferes with growth, nutrient and water relations, photosynthesis, assimilate partitioning and ultimately cause a significant reduction in crop yields (Farooq et al., 2009b; Praba et al., 2009). The plant response to drought stress generally varies from species to species depending on plant growth stage and other environmental factors (Demirevska et al., 2009). Reduced absorption of photosynthetically active radiations, impaired radiation use efficiency and decreased harvest index are the major yield reducing factors under limited supply of soil moisture (Earl and Davis, 2003). Plants show certain changes in their growth patterns and physiological process to cope with the drastic effects of drought stress (Duan et al., 2007).

Growth and development of the plants is also greatly affected by the series of morphological, biochemical and physiological changes resulted by high temperature stress (Wahid et al., 2007). At Present, heat shocks due to the rising atmospheric temperatures are becoming one of the major limiting factors to crop productivity around the globe. This rising temperature may cause a change in the growing periods and the distribution of the agricultural crops (Porter, 2005). High temperature stress may cause severe damage to the proteins, disturb their synthesis, inactivate major enzymes and damage membranes. Heat stress could also have major effects on the process of cell divisions (Smertenko et al., 1997). All these damages can seriously limit the plant growth and also favor the oxidative damage. In addition to all this brief exposure to the high temperature during the seed filling can result in accelerated filling and will finally result in poor quality and reduction in the yield. Here, we reviewed the basic responses of crop plants to drought and heat stress along with the management options which can be adopted to minimize the harmful effects of these abiotic stresses.

## Morphological Responses

### Growth

#### Drought

The initial effect of drought on the plants is the poor germination and impaired seedling establishment. Various studies have reported the negative impacts of drought stress on germination and seedling growth (Kaya et al., 2006; Farooq et al., 2009b). The reduction in germination potential, early seedling growth, root and shoot dry weight, hypocotyl length, and vegetative growth have been reported in important field crops including, pea (*Pisum sativum* L.), alfalfa (*Medicago sativa* L.), and rice (*Oryza sativa* L.) under drought stress (Okcu et al., 2005; Manikavelu et al., 2006; Zeid and Shedeed, 2006). Plant growth is mainly accomplished by cell division, enlargement, and differentiation. Drought impairs mitosis and cell elongation which results in poor growth (Hussain et al., 2008). Drought limits the process of cell growth mainly due to the loss of turgor (Taiz and Zeiger, 2006). Water limiting conditions results in impaired cell elongation mainly because of the poor water flow from xylem to the nearby cells (Nonami, 1998). Number of leaves and the size of individual leaf are also reduced under the drought conditions. The expansion of the leaf normally depends upon the turgor pressure and the supply of assimilates. Reduced turgor pressure and slow rate of photosynthesis under drought conditions mainly limit the leaf expansion (Rucker et al., 1995). Fresh and dry weights are also severely reduced under the water limiting conditions (Zhao et al., 2006). Plant height, leaf size, and the stem girth were significantly reduced under the water limiting conditions in maize (Khan et al., 2015). In another study, Kamara et al. (2003) reported that the biomass accumulation in maize was significantly reduced under drought conditions imposed at various growth stages.

#### Heat Stress

Under the tropical climates the excessive radiations and elevated temperatures are another major limiting factor to plant growth and development. High temperatures may cause scorching of the twigs and leaves along with visual symptoms of sunburn, leaves senescence, growth inhibition and discoloration of fruits and leaves (Ismail and Hall, 1999; Vollenweider and Gunthardt-Goerg, 2005). Elevated temperatures can reduce the germination potential of the seeds and, thus, results in poor germination and stand establishment. Adverse effects of high temperature on cereal crops vary with the timing, duration, and sternness of the heat stress (Fahad et al., 2016b). High temperature stress reduced number of spikes and number of florets per plant in rice and seed-set in sorghum was also negatively affected under similar conditions (Prasad et al., 2006; Fahad et al., 2016b). Inside a floret, anthers, and pollens were more susceptible to high temperature than ovules. Under high temperature (≥30°C), floret sterility has been correlated with diminished anther dehiscence, poor shedding of pollens, poor germination of pollen grains on the stigma, decreased elongation of pollen tubes and reduced *in vivo* pollen germination (Fahad et al., 2015b, 2016b). A significant reduction in the growth and net assimilation rate was observed in maize and sugarcane (*Saccharum officinarum* L.) under heat stress (Ashraf and Hafeez, 2004; Wahid and Close, 2007). Ebrahim et al. (1998) reported a significant reduction in the inter-nodal length and biomass accumulation along with early leaf senescence in sugarcane under heat stress.

### Yield

#### Drought

Yield is basically the complex integration of the different physiological processes. Most of these physiological processes are negatively affected by the drought stress. The negative impacts of drought on the yield mainly depend upon the severity of the stress and the plant growth stage. Significant yield losses have been reported in major field crops due to drought stress (**Table 1**). The drought induced at the pre-anthesis stage shortened the time to anthesis while that applied after anthesis reduced the period of grain filling in cereals (Estrada-Campuzano et al., 2008). The process of the grain filling in cereals is controlled by four major enzymes, i.e., Sucrose Synthase, Starch Synthase, Starch Branching Enzyme, and Adenosine Diphosphate Glucose Pyrophosphorylase (Taiz and Zeiger, 2006). A decreased activity of these enzymes has been reported under the drought conditions which have a negative impact on the yield of major cereal (Ahmadi and Baker, 2001). The exposure of plants to drought stress at the flowering may result in complete sterility in pearl millet (*Pennisetum glaucum* L.) which is usually due to the disturbed assimilate movement to the developing ear (Yadav et al., 2004).

**Table 1:** Yield losses in some major crops caused by drought and heat stress.

Crop species	Stress	Yield losses (%)	Reference
Maize (*Zea mays* L.)	Drought	63–87	Kamara et al., 2003
	Heat	42	Badu-Apraku et al., 1983
Wheat (*Triticum aestivum* L.)	Drought	57	Balla et al., 2011
	Heat	31	Balla et al., 2011
Rice (*Oryza sativa* L.)	Drought	53–92	Lafitte et al., 2007
	Heat	50	Li et al., 2010
Chickpea (*Cicer arietinum* L.)	Drought	45–69	Nayyar et al., 2006
Soybean (*Glycine max* L.)	Drought	46–71	Samarah et al., 2006
Sunflower (*Helianthus annuus* L.)	Drought	60	Mazahery-Laghab et al., 2003

Drought induced reduction in the yield might be due to various factors such as decreased rate of photosynthesis (Flexas et al., 2004), disturbed assimilate partitioning (Farooq et al., 2009b), or poor flag leaf development (Rucker et al., 1995). The exposure of maize to drought conditions at the tasseling stage resulted in a significant yield loss (Anjum et al., 2011). Similarly, a significant reduction in the boll production and the abortion of the produced bolls was recorded in cotton under drought conditions which ultimately affected the lint yield (Pettigrew, 2004). A significant reduction in the grain yield of barley (*Hordeum vulgare* L.) was also observed under drought conditions mainly because of less number of fertile tillers and grains along with less 1000 grain weight (Samarah, 2005). The exposure of pigeon pea (*Cajanus cajan* L.) to drought stress at the flowering stage caused over 50% reduction in the seed yield (Nam et al., 2001).

#### Heat Stress

In temperate regions the high temperature shocks during the reproductive phase can cause substantial reduction in the yield of major cereals. The quality of the final produce in cereals and oilseed crops are also negatively affected by heat stress as it reduces the oil, starch, and protein contents substantially (Wilhelm et al., 1999; Maestri et al., 2002). Ferris et al. (1998) reported a significant decline in the grain weight and total number of grains in wheat under elevated temperatures. Temperature stress reduced rice yield by reducing the performance of different rice growth and yield traits. Fahad et al. (2016a) reported that tillering was very sensitive to elevated night temperature in rice. The grain weights for a rice cultivar are nearly stable in a stress-free environment (Mohammed and Tarpley, 2010), however, under high night temperature, a decrease in individual grain weight resulted in significant reduction in rice grain production per unit area (Fahad et al., 2016a). Heat stress caused substantial yield reductions in common beans (*Phaseolus vulgaris* L.) and peanut (*Arachis hypogea* L.) (Vara Parasad et al., 1999; Rainey and Griffiths, 2005). A major effect of the heat stress is commonly noticed in tomato (*Lycopersicum esculentum* Mill.) as it influences meiosis, fertilization, and growth of fertilized embryo ultimately causing a noticeable reduction in the yield (Camejo et al., 2005).

Drought and heat stress causes significant reductions in growth and yield of several important crops; however, the extent of damage depends upon crop growth stage and severity of the stress. In general, the reproductive phase is more sensitive to the stresses causing a substantial reduction in the yield.

## Physiological Responses

### Water and Nutrient Relations

#### Drought

Water relations are influenced by certain factors including the leaf water potential, leaf and canopy temperature, transpiration rate, and stomatal conductance. Exposure to drought stress disturbs all these factors in plants however, stomatal conductance is affected the most (Farooq et al., 2009b). A significant reduction in the leaf water potential and transpiration rate was observed under the drought conditions which ultimately increased the leaf and canopy temperature (Turner et al., 2001). Another important feature for plant physiological regulation is water use efficiency which is the ratio of the dry matter accumulated to the water consumed (Monclus et al., 2006). Efficient cultivars of wheat have higher water use efficiency under drought stress (Abbate et al., 2004). This improvement in the water use efficiency is mainly due to the accumulation of the dry matter by consuming less amount of water due to the closing of stomata and less rate of transpiration. A reduced water use efficiency was observed in potato (*Solanum tuberosum* L.) when exposed to an early season water shortage and it ultimately resulted in poor biomass accumulation and yield (Costa et al., 1997).

Drought stress greatly impacts the nutrient relations of the plants. Many important nutrients including, nitrogen, silicon, magnesium, and calcium are uptaken by roots along with water, the drought conditions limit the movement of these nutrients via diffusion and mass which leads to retarded plant growth (Barber, 1995). Plants increase the length and surface area of roots and change their architecture in order to capture the less mobile nutrients (Lynch and Brown, 2001). The soil moisture deficit at times reduce the growth of the roots and, hence, reduce the uptake of the less mobile nutrients such as phosphorus (Garg, 2003). Root–microbe interactions also play an important role in nutrient relations of a plant. The impaired carbon and oxygen flux to the nodules coupled with N accumulation under drought stress inhibited N fixing ability of certain legumes (Ladrera et al., 2007). Composition and activity of the soil microbial colonies are negatively affected by the soil water deficit which eventually disturb the plant nutrient relations (Schimel et al., 2007). The responses to the mineral uptake under moisture stress varies across the crop species. In general, N uptake is increased, P uptake is declined and potassium remains unaffected under drought conditions. However, nutrient relations become more complicated due to interactive effects of different nutrients on each other and overall plant physiology. This aspect requires detailed research at a sophisticated molecular level.

#### Heat Stress

Plant water status is of prime importance under changing temperature conditions. Generally, the plants try to stabilize their tissue water content irrespective of temperature changes when the ample quantity of moisture is available; however, the temperature increase proves fatal under limited supply of water (Machado and Paulsen, 2001). Unfortunately, heat stress commonly coincides with the water scarcity under field condition especially in tropical and sub-tropical environments (Simoes-Araujo et al., 2003). Rapid reduction in leaf tissue water contents was observed in sugarcane on exposure to high temperature despite of the fact that the ample quantity of water was available in the soil (Wahid and Close, 2007). It shows that heat stress could also have a negative impact on the root conductance. Similar reduction in water content and root conductance has also been reported in tomato under heat stress (Morales et al., 2003). Generally, water loss under heat stress is more during day time mainly due to increased rate of transpiration, ultimately impairing certain important physiological processes in plants. Heat stress also reduces the number, mass and growth of the roots which ultimately limit the supply of water and nutrients to the above ground parts of the plant (Wahid et al., 2007; Huang et al., 2012).

Very little information is available regarding the direct impact of heat stress on the nutrient relations of crops (Basirirad, 2000; Rennenberg et al., 2006). Activity of the major enzymes like nitrate reductase involved in the nutrient metabolism can also be significantly reduced under high temperature stress (Klimenko et al., 2006). Reduction in nutrient uptake under heat stress might be due to various factors such as reduced root mass and nutrient uptake per unit root area (Basirirad, 2000). Overall, drought and heat stresses affect nutrient cycling, uptake and availability to plants by hampering different physiological functions of plants (**Table 2**).

**Table 2:** Effect of drought and heat stress on crop nutrients.

Stress	Process affected	Nutrient loss
Drought	Soil integrity by erosion	Loss of all mineral nutrients
Drought and heat	Transpiration driven mass flow	Mobile nutrients such as Ca, Mg, Si, nitrates, and sulfates
Drought and heat	Root growth	Almost all nutrients especially P and K
Drought and heat	Biological nitrogen fixation	N
Drought and heat	Soil microbial activity	N
Heat	Plant phenology	N, P, K
Heat	Nitrate reductase activity	N

### Photosynthesis

One of the key physiological phenomena affected by the drought and heat stress in plants is photosynthesis (Farooq et al., 2009b). It is mainly affected due to reduced leaf expansion, improper functioning of the photosynthetic machinery and leaf senescence (Wahid et al., 2007). Stomatal closure under drought reduces the CO_2_ availability which makes plant more susceptible to photo damage (Lawlor and Cornic, 2002). The reduced moisture availability induces negative changes in photosynthetic pigments, damages the photosynthetic machinery (Fu and Huang, 2001) and impairs the performance of important enzymes (Monakhova and Chernyadev, 2002) causing considerable loses in plant growth and yield. Similarly, the heat stress also impairs the process of photosynthesis by disturbing the photosynthetic pigments (Camejo et al., 2006), reducing activity of photosystem II (Camejo et al., 2005) and impairing the regeneration capacity of RuBP (Wise et al., 2004). Described below are some of the key effects of heat and drought stress on photosynthetic process.

#### Photosynthetic Pigments

##### Drought

Drought damages the photosynthetic pigments and the thylakoid membranes (Anjum et al., 2011). The reduction chlorophyll contents under drought conditions has also been reported (Din et al., 2011). However, some studies have reported an increase in chlorophyll contents in cereals under moisture stress (Estill et al., 1991). It seems to depend on crop and cultivar type. For instance, chlorophyll contents in some cultivars of black gram [*Vigna mungo* (L.) Hepper] were increased while in some others they were decreased under moisture stress (Ashraf and Karim, 1991). This varied behavior was attributed to the variation in the activities of enzymes involved in chlorophyll biosynthesis. It has been reported that concentration of chlorophyll *a* was higher as compared to chlorophyll *b* in drought stressed plants (Jain et al., 2010). A decrease in the chlorophyll *a*/*b* ratio was reported in *Brassica* species under drought conditions (Ashraf and Mehmood, 1990).

##### Heat Stress

Exposure to high temperature usually results in reduction in chlorophyll biosynthesis (Dutta et al., 2009). Reduced accumulation of the chlorophyll in the plants may be due to either decreased biosynthesis of the chlorophyll or due to its increased degradation or combined effect of both under high temperature stress. The chlorophyll biosynthesis inhibition under high temperature stress is actually attributed to the deactivation of various enzymes (Dutta et al., 2009). For instance, the activity of 5-aminolevulinate dehydratase, an important enzyme in the pyrrole biosynthesis pathway, decreased significantly in wheat under heat stress (Mohanty et al., 2006). Chlorophyll biosynthesis in cucumber (*Cucumis sativus* L.) was reduced by 60% at 42°C mainly due to the inhibition of the synthesis of the 5-aminolevulinate at high temperature regimes (Tewari and Tripathy, 1998). A 70% reduction in biosynthesis of the protochlorophyllide was also observed under elevated temperatures (Karim et al., 1999). Heat stress caused more accelerated degradation of chlorophyll *a* and *b* in developed leaves (Karim et al., 1999). These impacts on the pigments and other photosynthetic apparatus are also supposed to be associated with oxidative damage (Guo et al., 2006). An increased chlorophyll *a*/*b* ratio was noticed along with a considerable decrease in chlorophyll to carotenoid ratio in heat tolerant cultivars of tomato and sugarcane plants (Camejo et al., 2005). It shows that a change in the pigments ratio has also a role in the tolerance against heat shocks.

#### Photosynthetic Process

##### Drought

The first and foremost response of almost all the plants to moisture stress is stomatal closure in order to avoid the water loss through transpiration. The stomatal closure may be in the response of the reduced leaf water potential (Ludlow and Muchow, 1990) or to a decreased level of humidity in the atmosphere (Maroco et al., 1997). Stomatal closure checks CO_2_ intake which leads to oxidative damage and no assimilation. The stomatal closure also increases the heat dissipation in leaves (Yokota et al., 2002). Interestingly, stomatal regulation is more affected by the soil moisture status than the leaf water content, it might be the reason that stomata responds to the ABA which is produced by the roots under drought conditions (Turner et al., 2001). However, stomatal responses are highly variable under drought conditions across the plant species (Lawlor and Cornic, 2002).

Photosynthesis is limited by reduced stomatal conductance under light drought, however, impaired functioning of the Rubisco becomes the major factor affecting photosynthesis (Bota et al., 2004). Water shortage causes shrinkage of the cell due to which a decrease in the cellular volume takes place, as a consequence the cellular material become more viscous which leads to denaturation of the proteins (Hoekstra et al., 2001). Elevated levels of solutes in the cytoplasm may also result in ion toxicity having a severe impact on the activity of the enzymes involved in photosynthesis and other plant processes (Hoekstra et al., 2001). The concentration of the Rubisco enzyme in leaves depends upon the rate at which it is synthesized and degraded. It remains quite stable even under acute water shortage because of having a half-life of several days (Hoekstra et al., 2001). However, major damage is caused by the decreased synthesis of the Rubisco due to the decrease in its small subunits (Vu et al., 1999). Binding of inhibitors like 2-Carboxyaribinitol 1-Phosphate to the catalytic site of Rubisco is also common under drought stress which affects enzyme activity. Similarly, other important enzymes involved in photosynthesis are also negatively affected by drought and heat stresses (**Table 3**). The decreased phosphorylation and impaired ATP synthesis has been reported as the major factors limiting photosynthesis under mild drought (Lawlor and Cornic, 2002). The reduced production of nicotinamide adenine dinucleotide phosphate under the drought conditions results in down regulation of the non-cyclic electron transport chain which reduces the ATP synthesis.

**Table 3:** Activity of photosynthetic enzymes in some field crops as influenced by drought and heat stress.

Crop	Stress	Enzyme	Activity	Reference
Alfalfa (*Medicago sativa* L.)	Drought	Rubisco	Unchanged	Medrano et al., 1997
Tobacco (*Nicotiana tabacum* L.)	Drought	Rubisco	Reduced	Parry et al., 2002
	Heat	Rubisco	Reduced	Crafts-Brandner and Salvucci, 2002
Maize (*Zea mays* L.)	Drought	PEPCase	Increased	Jeanneau et al., 2002
	Heat	Rubisco-activase	Reduced	Crafts-Brandner and Salvucci, 2002
Sugarcane (*Saccharum officinarum* L.)	Drought	Phosphoenol pyruvate	Reduced	Du et al., 1996
		carboxylase (PEPCase), PPDK	
Cotton (*Gossypium hirsutum* L.)	Heat	Rubisco	Reduced	Crafts-Brandner and Salvucci, 2002
Wheat (*Triticum aestivum* L.)	Heat	Rubisco	Reduced	Salvucci and Crafts-Brandner, 2004

##### Heat Stress

Light dependent chemical reactions taking place in the thylakoid and the C metabolism taking place in the stroma are the main sites of damage as a result of the high temperature stress. Increased temperature of the leaf and photon flux density effects the thermo-tolerance adjustment of the PSII (Crafts-Brandner and Salvucci, 2002). The PSII is very much responsive to temperature and its activity is greatly influenced and even partially terminated under high temperature stress (Camejo et al., 2005). Oxygen evolving complex is also subjected to serious damage under higher temperature which may result in imbalanced flow of electrons to the acceptor site of PSII (De Ronde et al., 2004). The D_1_ and D_2_ proteins are also subjected to denaturation under higher temperature (Rivas and Barber, 1997).

Different components of PSII were damaged under heat stress in wheat and barley (Sharkova, 2001; Toth et al., 2005). Similarly, photosynthesis in cotton was limited due to disruption in electron transport chain and the reduced RuBP regeneration capacity (Wise et al., 2004). Under high temperatures, the PSII stromal enzymes and chloroplast are very much stable and the PSII driven electron transport chain is activated (Bukhov et al., 1999). In a recent study, Fahad et al. (2016a) reported that high day as well as night temperatures reduced the photosynthetic activities of two rice cultivars (IR64 and Huanghuazhan) significantly. The reduction in photosynthesis was ascribed to the damage to chlorophyll pigments, decline in leaf nitrogen contents, blockage of PSII reaction center and electron flow, decreased quantum efficiency (Fv/Fm) and down-regulation of PSII photochemistry.

Under high temperature stress the synthesis of starch and sucrose is greatly affected due to a reduction in the activities of important enzymes such as adenosine diphosphate-glucose pyrophosphorylase, sucrose phosphate synthase, and invertase (Vu et al., 2001). Net photosynthesis in many plant species is inhibited due to reduction in the activation state of the CO_2_ binding enzyme, Rubisco (Crafts-Brandner and Salvucci, 2002; Morales et al., 2003). Although the catalytic activity of Rubisco increases with rising temperature, its low affinity toward CO_2_ and capability of binding with O_2_ limits the increase in net photosynthesis rate (Crafts-Brandner and Salvucci, 2002). Despite of all these negative effects of high temperature on photosynthesis, the optimum temperature requirements for photosynthesis are expected to rise with elevating concentration of CO_2_ in the atmosphere.

### Assimilate Partitioning

#### Drought

Drought disturbs the balance of assimilates as most of them are translocated to the roots in order to improve water uptake (Leport et al., 2006). The export of assimilates from source to sink generally depends upon the rate of photosynthesis and the sucrose concentration in leaves (Komor, 2000). Drought impairs the process of photosynthesis and decrease the sucrose content which ultimately reduce the export rate from source to sink (Kim et al., 2000). Drought also limits the ability of the sink to utilize the incoming assimilates efficiently (Zinselmeier et al., 1999). Moreover, the activity of acid invertase is negatively affected which disrupts the phloem loading and unloading. In this way, dry matter partitioning is badly effected under moisture stress.

#### Heat Stress

Reduction in the activities of source and sink takes place under heat stress which greatly effects the growth and ultimately the economic yield (Taiz and Zeiger, 2006). However, a considerable variation was reported among different wheat genotypes regarding assimilate partitioning under heat stress (Yang et al., 2002). Dinar and Rudich (1985) reported that the transport of the C to the apex was significantly reduced in two heat sensitive cultivars of tomato. Wardlaw (1974) examined the response of source, sink and transport pathway to high temperature stress in wheat and found that the photosynthesis rate was optimum at 20–30°C, however, an abrupt decline was noticed above 30°C. Loading of assimilates from the flag leaves also followed the same trend (Wardlaw, 1974). However the movement within the stem was found independent of the temperature from 1 to 50°C. It was concluded that the effect of heat stress on assimilate partitioning in wheat was indirectly related to the abnormal behavior of source and sink in addition to the decreased photosynthesis rate. From such observations we can suggest that the improvement of the mobilization efficiency of assimilates from leaves and other plants parts can be an important strategy for improving grain filling in the cereals.

## Oxidative Damage: A Common Response

Oxidative damage is usually a subsequent stage of most of the abiotic stresses in plants. Exposure of plants to drought stresses initially causes oxidative damage by the formation of ROS. These ROS pose serious threat to the cell functioning by damaging lipids and proteins. In pea, the lipid and protein peroxidation was increased by four times under drought stress as compared with normal conditions (Moran et al., 1994). The ROS are mainly produced in the chloroplast (Reddy et al., 2004), however, reaction of oxygen with the components of electron transport chain in mitochondria also results in the generation of ROS (Moller, 2001). The mechanisms involved in the generation of the ROS can be either enzymatic or non-enzymatic (Apel and Hirt, 2004). The production of the ROS has also been reported under high temperature stress (Liu and Huang, 2000; Wahid et al., 2007).

In order to cope with the oxidative stress, plants usually rely on the antioxidant defense which can be either enzymatic or non-enzymatic. Enzymatic defense is usually considered as the most effective (Farooq et al., 2008). Major enzymes involved in this system are SOD, GR, POD, and CAT (Farooq et al., 2009b). Beside these enzymes, certain carotenoids and glutathione can also play part in the antioxidant system as non-enzymatic components. The enzymes such SOD, POD and CAT either directly scavenge the ROS or protect plants indirectly by managing non-enzymatic defense (Anjum et al., 2011). In response to ROS, an increased content of malondialdehyde has been reported which is a pure indicator of drought induced oxidative damage (Moller et al., 2007). Therefore, maintenance of the higher levels of the anti-oxidants can be a good strategy by the plants to counter the negative effects of ROS (Sharma and Dubey, 2005). Phytohormones are also natural defense molecules in plants maintains higher levels of the anti-oxidants under stress. They help plants to acclimatize to varying environments by mediating growth, development, source/sink transitions, and nutrient allocation (Fahad et al., 2015a).

## Management Options

The genetic improvements in combination with the proper cultural practices are considered important in managing the abiotic stresses (Wahid et al., 2007). Several cultural practices has been long practiced to cope with abiotic stresses; however, the use of the genetic tools for this purpose is relatively recent inclusion. In past, major focus of the breeders has remained on the development of high yielding varieties and no doubt those varieties performed best under the non-stressed environment. However, on the face of changing climatic conditions where the plants are more prone to abiotic stress, the emphasis should also be given to breeding for stress tolerance. In the recent past, research has been started to improve the stress tolerance in the plants by using the conventional and molecular breeding approaches (Farooq et al., 2009b). Major management strategies have been discussed below in detail.

### Conventional Breeding

#### Drought

Plant breeding using typical old techniques has proved very handy for the identification of stress-tolerant genetic traits in various crops and cultivars and the transfer of those traits into the cultivars having good agronomic performance (Ashraf, 2010). A significant progress has been made by the international research institutes to develop cultivars with noticeable drought tolerance (**Table 4**). One good example is the breeding effort started by CIMMYT for improving the tolerance in the maize against drought and common diseases. These hybrids were found best under drought in terms of overall plant growth and the economic yield. In 2006, a drought resistant cultivar of maize, Obatanpa GH, was developed by Crops Research Institute, Ghana in collaboration with CIMMYT and IITA which helped for food security in drought-hit regions (Badu-Apraku and Yallou, 2009). Similarly, IITA has also developed 16 inbred lines of maize having a certain degree of tolerance against drought (Badu-Apraku and Yallou, 2009).

**Table 4:** Drought tolerant cultivars of some important field crops developed by different research institutes through conventional breeding.

Research Institute	Crop	Cultivar/line	Reference
International Center for Tropical Agriculture (CIAT)	Common beans	SEA-5	Singh et al., 2001
	Common beans	SEA-13	Singh et al., 2001
	Common beans	A-195	Singh et al., 2007
International Center for Agricultural Research in the Dry Areas (ICARDA)	Chickpea	Flip 87-59C	Singh et al., 1996
	Barley	Giza-126	Noaman et al., 1995
International Crops Research Institute for the Semi-Arid Tropics (ICRISAT)	Peanut	ICGV-87354	Reddy et al., 2001
Montana Agricultural Research Station, United States	Wheat	Willow Creek	Cash et al., 2009
Colorado Agricultural Experiment Station, United States	Wheat	Ripper	Haley et al., 2007
	Wheat	Prairie Red	Quick et al., 2001
Nebraska Agricultural Experimental Station, United States	Wheat	NE01643	Baenziger et al., 2008
Agricultural Research Station, Giza, Egypt	Barley	Giza-2000	Noaman et al., 2007
International Institute of Tropical Agriculture (IITA)	Maize	16 inbred lines	Badu-Apraku and Yallou, 2009

Wheat is an important cereal crop around the globe and according to an estimate nearly 50% of the area under wheat cultivation is subjected to periodic drought. At CIMMYT, a diploid wild relative of bread wheat (*Aegilops tauschii*) was crossed with a tetraploid (*Triticum turgidum*) to produce a hexaploid having a significant tolerance against major abiotic stresses (Valkoun, 2001). Similar efforts are also in progress in IRRI, Philippines for the development of drought tolerant rice cultivars using classic breeding approaches. It will further improve the adoption of direct-seeded rice. Plant breeders and researchers at ICARDA and ICRISAT are also working to develop the drought tolerant cultivars of major cereals and leguminous crops (Ashraf, 2010).

### Heat Stress

An easiest approach to develop heat tolerant cultivars is the examination of the breeding material under the hot target conditions and identification of the lines showing better performance (Ehlers and Hall, 1998). Different morpho-physiological traits are used as indicators of heat tolerance in identifying better performing varieties (**Table 5**). In general, the tolerance of the plant to heat stress is characterized by minimal damage to photosynthetic machinery and increased biosynthesis of the protective compounds (Bita and Gerats, 2013). Photosynthesis and reproductive phase of plant growth are highly sensitive to high temperature stress. So, a heat tolerant variety in this regard should have a better photosynthetic rate, membrane thermo stability and fruit setting under high temperature (Nagarajan et al., 2010). Some other indirect parameters used for selection include, the grain filling duration and grain weight (Yang et al., 2002; Sharma et al., 2008). Setimela et al. (2005) developed heat tolerance index to evaluate the recovery potential after heat shock as an important tolerance indicator. Although it is an easy criteria, its effectiveness for wide range of crops is yet questionable.

**Table 5:** Desirable plant traits for heat tolerance.

Trait	Reference
Canopy temperature depression	Reynolds et al., 1994
Thylakoid membrane stability	Kumar et al., 2012
Ability to stay green	Kumar et al., 2010
Waxy leaves	Richards, 1996
Chlorophyll content	Yang et al., 2002
Stomatal conductance	Reynolds et al., 1994
Photosynthetic rate	Nagarajan et al., 2010
Grain filling duration	Yang et al., 2002
Better fruit setting	Nagarajan et al., 2010
Grain yield	Yang et al., 2002

Conventional breeding is a nice approach, however, genetic variation in the existing germplasm is very limited and it takes long time to screen and test the existing genotypes before starting the breeding programs.

### Modern Breeding

#### Drought

Drought tolerance in the plants is a complex phenomenon being controlled by a large number of minor genes and loci on chromosomes having those genes called as QTL (Mohammadi et al., 2005). The Exploitation of the genetic variation among the existing cultivars for stress tolerance can be either done by natural selection under stressful environment or by QTLs mapping followed by marker assisted selection approach (Ashraf et al., 2008). Mapping of the QTLs basically helps in the assessment of total number of genes, their location and action pattern. A major problem associated with the selection of a proper QTL for the drought tolerance is a high degree interaction between QTL and environment (Tuberosa and Salvi, 2006). Therefore, once a QTL is identified for drought tolerance, isogenization is mandatory for its proper characterization (Salvi and Tuberosa, 2005). Mapping of the QTLs for the traits related to drought tolerance has been done in variety of crop species as enlisted in earlier reviews (Ashraf, 2010; Farooq et al., 2014; Lata et al., 2015). In cotton, a set of 33 QTLs was identified under water shortage conditions by using F_3_ families of a cross between *Gossypium barbadence* and *Gossypium hirsutum* (Saranga et al., 2001). Out of those 33 QTLs, five were related to physiological traits, 11 to plant productivity and 17 to fiber quality. Recently the NILs had also been developed by shifting the QTLs for yield and other traits among *G. barbadence* and *G. hirsutum* (Levi et al., 2009). A large number of QTLs associated with drought tolerance has also been identified in rice (Lafitte et al., 2007). Another study identified 36 related to root growth and five related to osmotic adjustment in rice (Zhang et al., 2001). The *Deeper Rooting 1 (DRO1)* has proven to be a very useful QTL for drought tolerance as it increases root length in plants. For instance, Uga et al. (2013) reported significant yield improvement in shallow rooted rice cultivars after the introduction of *DRO1.*

After the identification of the proper QTLs, the next important part is their manipulation in order to develop drought tolerant cultivars. Steele et al. (2006) improved the root morphological characters in an Indian rice cultivar “Kalinga III” through marker assisted back crossing approach where a japonica cultivar “Azucena” from Philippines was used as donor. Five fragments on different chromosomes were selected for introgression. Among them four contained the QTLs for improved root morphology while one had QTL for aroma. Steele et al. (2007) evaluated four NILs under drought and observed improvement in grain and straw yields. Certain QTLs responsible for drought tolerance has also been identified in pearl millet by the breeders at ICRISAT (Serraj et al., 2004). The introgression lines developed by the marker assisted back crossing of the identified QTLs in the drought sensitive variety resulted in improved yield and better drought tolerance (Serraj et al., 2005). The stay green character under water limiting condition in sorghum was also improved by this approach (Harris et al., 2007). All these studies clearly show that QTL mapping and marker assisted breeding can play a vital role in improving the crop tolerance against drought stress. Although the achievements made so far seems simple, correct identification of the QTLs and proper application of marker assisted techniques is a complicated and expensive task.

#### Heat Stress

Modern breeding approaches involving QTL mapping have not been used extensively for heat stress tolerance. The QTLs related to different traits involved in heat tolerance such as grain filling duration and leaf senescence have been identified in wheat (Mason et al., 2010). Farooq et al. (2011) also listed several QTLs identified for heat tolerance in wheat during reproductive stage. The QTLs related to grain filling duration in wheat under high temperature were identified on chromosome number 1B and 5A (Yang et al., 2002). A set of nine QTLs for tillering and three QTLs for stay green character has also been identified in wheat (Kumar et al., 2010; Li et al., 2010). Moreover, a set of four QTLs in *Arabidopsis* and 11 QTLs in maize were identified playing an important role in thermo-tolerance (Frova and Sari-Gorla, 1994).

A wide range of markers associated with the QTLs of heat tolerance has been identified, however, their actual role in marker assisted selection is very limited (Kumar et al., 2013). However, the simple sequence repeat markers linked with different heat tolerance characters were used recently in marker assisted selection among 25 wheat genotypes for heat tolerance (Sadat et al., 2013). The research work for identifying markers linked to heat tolerance has great scope and requires more effort.

### Transgenic Approach

#### Drought

Transgenic approaches involve modifications in the qualitative as well as the quantitative traits through transfer of desired genes (Ashraf, 2010). The major emphasis has been on the engineering of the genes which encode growth regulators, compatible solutes, and antioxidants involved in stress tolerance. The genes encoding two enzymes (choline mono oxygenase and beta aldehyde dehydrogenase) responsible for glycine betaine expression in higher plants have been successfully engineered to develop drought tolerant crops (Zhang et al., 2008). An inbred line of maize (DH4866) has also been produced by transferring a beta gene from *Escherichia coli* resulting in improved glycine betaine production and ultimately better drought tolerance (Quan et al., 2004). Similarly, the genes encoding the enzyme involved in the biosynthesis of another important osmolyte, proline, have been engineered in various crops including, soybean [*Glycine max* (L.) Merr.] and tobacco (*Nicotiana tabacum* L.) (Ronde et al., 2004; Gubis et al., 2007). The genes regulated by *DREB* and *AREB* protein are also being studied for drought stress tolerance in several crops (Singh and Laxmi, 2015). Recently, Kudo et al. (2017) reported that double overexpression of *DREB1A and OsPIL1* genes improved drought tolerance in transgenic plants. Similarly, a large number of genes related to NAC family have been identified for stress tolerance in sugarcane (Ramaswamy et al., 2017).

Different types of the antioxidants are produced by the plants which play an important role in improving tolerance against the oxidative damage (Sunkar et al., 2006). Genes involved in the production and expression of SOD has been engineered and to produce drought tolerant transgenic alfalfa, potato and rice (Perl et al., 1993; McKersie et al., 1996; Wang et al., 2005). Similarly, transgenic tobacco has also been produced showing the over expression of ascorbate peroxidase and mono dehydro-ascorbate reductase (Eltayeb et al., 2007). Accumulation of the LEA proteins also plays an important role in drought tolerance (Gosal et al., 2009). The LEA proteins help plants in maintaining the cell membrane structure and ionic balance under drought stress (Browne et al., 2002). In the recent years, efforts have been put forth to engineer the genes involved in the production of LEA proteins. Transgenic lines of wheat, sorghum, and rice have been developed by transferring such genes to improve drought tolerance (Xu et al., 1994; Cheng et al., 2002). The drought tolerance capacity of transgenic crops depends on the crop growth stage and intensity of stress (Reddy et al., 2004; Ashraf, 2010). Further research is required to produce more transgenic crops with higher yield potential.

#### Heat Stress

Significant progress has also been made in identification of the genes involved in various mechanisms of heat tolerance and their manipulation using various transgenic approaches (Zhang et al., 2001; Bonhert et al., 2006). Genetic manipulations for over-expression of SOD under heat stress has proved successful (Sairam and Tyagi, 2004). A transgenic tobacco plant showing a better photosynthetic activity under heat stress has been produced by alteration of the chloroplast membranes (Murakami et al., 2000). An enhanced tolerance against high temperature was reported in tobacco by the transfer of a gene *Dnak1* (Ono et al., 2001). The transgenic plants having better production of glycine betaine due to transfer a gene *(BADH)* showed more tolerance to heat stress (Yang et al., 2005). The improved tolerance against heat stress can also be achieved by over expression of the HSPs through genetic manipulations. A transgenic tobacco plant was produced by the transfer of *MT-sHSP* from tomato for better thermo-tolerance (Sanmiya et al., 2004). Similarly, *HSFs* and *DREB2A* genes have been identified to engineer heat tolerant transgenic plants (Ohama et al., 2017).

### Inducing Stress Resistance

#### Drought

Exogenous application of growth regulators and osmo-protectants at different growth stages can play an important role in inducing resistance against drought. A very important and short-term approach in this regard is seed priming which is a pre-sowing hydration of the seed in such a way that the germination metabolism is initiated but the emergence of radicle is avoided (Farooq et al., 2006). Seed priming has proved beneficial in improving the germination metabolism and early stand establishment of crops under normal and stress conditions (Farooq et al., 2007; Bajwa and Farooq, 2016). The priming of the rice seedlings with 5% polyethylene glycol and sodium chloride solutions significantly improved their performance under drought conditions (Goswami et al., 2013). Similarly, a 44% increase in the germination of wheat seeds was also recorded by seed priming under drought conditions (Ajouri et al., 2004). An improved performance of some wheat cultivars was reported under drought conditions after priming with potassium chloride (Eivazi, 2012). Priming of the wheat seeds with ascorbic acid resulted in improved drought resistance due to better accumulation of the proline which helped to maintain the tissue water content and membrane stability (Farooq et al., 2013b). The priming of the maize seeds with putrescine improved the tissue water content and total biomass accumulation under both water limiting and well-watered conditions (Hussain et al., 2013). Khan et al. (2015) reported that the seed priming with calcium chloride played a significant role in improving the performance of maize hybrids under water limiting conditions. A similar improvement in sunflower (*Helianthus annuus* L.) germination potential, growth and yield was reported by seed priming with potassium nitrate under drought conditions (Kaya et al., 2006). Seed priming with low concentration of allelopathic crop water extracts has also emerged as a beneficial tool to improve crop growth and yield under normal and stressful conditions (Farooq et al., 2013a; Bajwa and Farooq, 2016).

Another important approach for inducing resistance in plant against abiotic stresses is the exogenous application of the growth regulators. The application of the growth regulators helps plants to maintain a fair water balance and chlorophyll content under drought. Foliar applied gibberellic acid improved the stomatal conductance, rate of transpiration, and net photosynthesis in cotton under water limiting conditions (Kumar et al., 2001). Application of jasmonates in combination with brassinolides improved the drought tolerance of maize mainly due to better antioxidant defense (Li et al., 1998). A positive role of the brassinosteroids has been observed in inducing resistance against drought (Rao et al., 2002). Brassinolides were also found useful in improving the germination and seedling growth of the sorghum under water limiting conditions (Vardhini and Rao, 2003). The application of the brassinolides improved the performance of rice under drought and improved the CO_2_ assimilation and leaf water economy (Farooq et al., 2009a). Anjum et al. (2011) reported that the exogenous application of the brassinolides improved the performance of maize under drought conditions by improving the water relations and antioxidant defense. Application of salicylic acid improved the drought resistance in wheat by improving the activity of antioxidant enzyme catalase (Horvath et al., 2007). ABA plays an important role in drought stress tolerance and the development of different ABA analogs for ABA receptors have advanced its use in drought stress tolerance (Okamoto et al., 2013).

Exogenous application of osmo-protectants has also been used effectively to improve drought resistance in plants (Ashraf and Foolad, 2007). For instance, the application of glycine betaine can help plants in improving their performance under drought conditions (Hussain et al., 2008). It improves the stomatal conductance, photosynthetic rate, proline accumulation in plants (Ma et al., 2007). Similarly, the application of spermidine has also been fond beneficial in minimizing the harmful effects of drought in barley (Kubis, 2003). Moreover, the application of silicon improved drought tolerance by improving the water uptake in sorghum (Hattori et al., 2005). Other studies have also highlighted the potential of silicon application in improving the drought resistance in major crops including, wheat, rice, and sorghum mainly through improved root growth, stomatal conductance, photosynthetic rate, and antioxidant defense (Lux et al., 1999, 2003; Gong et al., 2005; Hattori et al., 2005).

#### Heat Stress

Relatively less research has been done in resistance induction against heat stress. However, the basic mechanism of using growth regulators, osmo-protectants, and other chemicals is same for drought and heat stress. Preconditioning of plants has proved very effective to combat the heat stress. For instance, preconditioned tomato plants showed better performance under the heat stress by making better osmotic and stomatal adjustments (Morales et al., 2003). Pre-sowing the seeds of pearl millet when exposed to higher temperature (42°C) resulted in better performance (Tikhomirova, 1985). The positive effect of calcium under thermal stress has been reported in certain cool season grasses as it maintains the antioxidant activity (Jiang and Huang, 2001). It has been shown that the exogenous application of calcium can play important role in inducing heat stress resistance in plants by better activity of antioxidant defense (Kolupaev et al., 2005). Wahid and Shabbir (2005) reported that treatment of barley seeds with glycine betaine resulted in better performance under heat stress through improved membrane stability, photosynthetic rate, and leaf water status. Similarly, an improved performance of tomato plants under heat stress was observed by the application of spermidine (Murkowski, 2001).

Further research is needed to explore the potential of seed priming and foliar application of growth regulators in a wide range of crops under heat stress. Moreover, the integrated approaches focusing the use of these techniques with genetic modifications may also be evaluated.

## Conclusion and Future Prospects

Abiotic stresses are important constraint limiting the crop productivity worldwide. Plants show a wide range of responses to drought and heat stresses which are mostly depicted by a variety of alterations in the growth and morphology of plants. Although drought and heat stress may cause negative effects on overall growth and development of the plants, the major phase being damaged is the reproductive growth. A mild stress at anthesis or grain filling phase can substantially reduce the crop yield. Other noticeable effects of these stresses are damaged photosynthetic machinery, oxidative damage, and membrane instability. Plants ability to with stand these stresses greatly varies from species to species. Recently, major achievements have been made in minimizing the negative effects of these abiotic stresses either by adopting the genetic approaches or by inducing the stress resistance. Despite of the major advances in the genetic approaches such as QTL mapping and transgenic approaches there is still a big room for improvement. For example, the genetic and environmental interactions are poorly understood. Similarly, QTLs identified for one background does not perform best under different other backgrounds. Similarly, issues are still present with the transgenic plants developed for combating with heat and drought stress. Most of the transgenic plants developed are not tested under field conditions therefore; their performance under the field conditions is yet a question mark. The use of conditional promoters driving gene expression at specific developmental stages, in specific tissues/organs and/or in response to specific environmental cues, circumvents this problem and will make possible the generation of transgenic crops able to grow under various abiotic stresses with minimal yield losses.

## Author Contributions

SF, AAB, and UN conceived the idea of the review and prepared the initial outline and wrote the first draft. SF, UN, and AAB had major and equal contribution in overall preparation of manuscript. SAA, SA, AF, AZ, SF, SeS, MI, HA, CW, DW, and ShS gathered the literature and contributed in writing the different sections. JH, WN, and SA provided the technical guidance and editing support.

## Conflict of Interest Statement

The authors declare that the research was conducted in the absence of any commercial or financial relationships that could be construed as a potential conflict of interest.

## Abbreviations

ABA: abscisic acid
AREB: abscisic acid-responsive element binding
ATP: adenosine triphosphate
C: carbon
Ca: calcium
CAT: catalase
CH_4_: methane
CIMMYT: International Maize and Wheat Improvement Center
CO_2_: carbon dioxide
DREB: dehydration-responsive element binding
DREB1A: dehydration-responsive element-binding 1A
DREB2A: dehydration-responsive element-binding protein 2A
DRO1: *Deeper Rooting 1*
GR: glutathione reductase
HSFs: heat shock transcription factors
HSPs: heat shock proteins
ICARDA: International Center for Agricultural Research in Dry Areas
ICRISAT: International Crop Research Institute for Semi-Arid Tropics
IITA: International Institute of Tropical Agriculture
IRRI: International Rice Research Institute
K: potassium
LEA: late embryogenesis abundant
Mg: magnesium
N: nitrogen
NILs: near isogenic lines
O_2_: oxygen
OsPIL1: phytochrome-interacting factor-like 1
P: phosphorus
POD: peroxidase
PS: photosystem
QTL: quantitative trait loci
ROS: reactive oxygen species
Rubisco: ribulose-1,5-bisphosphate carboxylase/oxygenase
RuBP: ribulose bisphosphate
Si: silicon
SOD: superoxide dismutase.
